# Hematological parameters of the blood count in a healthy population of pregnant women in the Northwest of Morocco (Tetouan-M’diq-Fnideq provinces)

**DOI:** 10.11604/pamj.2018.29.205.13043

**Published:** 2018-04-09

**Authors:** Saad Bakrim, Youssef Motiaa, Ali Ouarour, Azlarab Masrar

**Affiliations:** 1 Laboratory of Biology and Health, Abdelmalek Essaâdi University, Faculty of Science, BP 2121, Tetouan, Morocco; 2 Hematology Laboratory, Provincial Hospital Center, Mohammed VI Hospital, M’diq, Morocco; 3 Hematology Laboratory, Faculty of Medicine and Pharmacy, Mohammed V University, Rabat, Morocco, Central Hematology Laboratory, Ibn Sina University Hospital, Rabat, Morocco; 4 Anesthesia Reanimation Department, Ibn Sina University Hospital, Rabat, Faculty of Medicine and Pharmacy, Mohammed V University, Rabat, Morocco

**Keywords:** Reference intervals, blood count, hematology, pregnant women, trimester, Northwest Moroccan population

## Abstract

**Introduction:**

Numerous biological parameters are physiologically modified during normal pregnancy, in particular hematology. The knowledge of these modifications of the maternal body by biologists and clinicians allows the screening of possible anomalies. In Morocco, the reference values of the complete blood count test for pregnant woman are missing, as are those specific to different trimesters of pregnancy. The aim of this study is to look for the reference values for healthy pregnant women of the Northwest region of Morocco, to compare them to those of non-pregnant women (control) and to those of the literature.

**Methods:**

Blood samples were taken voluntarily from 3898 healthy pregnant women from 18 to 46 years old who presented themselves at the center of health Kalaa and at the service of gynecology obstetrics of the Provincial Hospital Center of M'diq (Morocco), for prenatal care. To establish the reference intervals of the CBC for non-pregnant women, a control group was constituted by 7035 healthy women from 18 to 50 years old selected according to the Moroccan law of blood donation. The CBC was measured on a Sysmex KX21N^®^ analyzer. For each sample a systematic blood smear was done to determine the leukocyte differential.

**Results:**

A statistically significant difference between the pregnant women and control group was noted (p < 0.05) for all the hematological parameters: red blood cells, hematocrit, hemoglobin, mean corpuscular volume, mean corpuscular hemoglobin, mean corpuscular hemoglobin concentration, leukocytes, neutrophils, basophils, eosinophils, lymphocytes, monocytes, platelets and mean platelet volume. So, the comparison of the averages established between the first, second and third trimester of pregnancy showed the existence of a significant variation with regard to all the parameters of the CBC test looked for (p < 0.001).

**Conclusion:**

The present study provides additional baseline data for basic hematological parameters in healthy pregnant Moroccan women and concluded that pregnancy in women has the tendency to alter some hematological indices. For these reasons, there is an interest to take these modifications into account for optimal maternal and fetal medical care.

## Introduction

The values of the complete blood count (CBC) parameters can vary according to numerous pre-analytical, analytical, pathological and physiological factors such as age, sex, height, environment, race, nutritional state, ethnic origin, lifestyle, biorhythms, consumption of tobacco, alcohol or medicine or still the pregnancy [1]. This last factor is associated with profound anatomical, physiological, biochemical and endocrine changes that affect multiple organs and systems. These changes are essential in helping woman adapt to the state of pregnancy and to aid fetal growth and survival. The hematologic system must adapt in a number of ways, such as provision of vitamins and minerals for fetal hematopoiesis (iron, vitamin B12, folic acid), which can exacerbate maternal anemia and preparation for bleeding at delivery and requires enhanced hemostatic function [2]. One of the most significant hematological changes for pregnant woman is physiologic anemia due to independent and uneven variations of plasmatic volume (+ 40%) and corpuscular volume (+15%) [3]. The phenomenon of hemodilution further contributes to a reduction in the rate of hematocrit (HCT) and hemoglobin (HGB), resulting in a false anemia. For pregnant woman, such a modification is physiological and proves the adoption of a different threshold for the definition of the pregnancy anemia. Concerning the hemoglobin and according to the Centers for Disease Control and prevention (CDC) in the United States, the HGB must be lower than 11.0g/dL in 1st and 3rd trimesters and lower than 10.5 g/dL in the 2nd one [4]. For the WHO, the threshold of anemia in pregnancy is a state in which the total circulating HGB concentration is less than 11g/dl; or HCT less than 33% at any time of the pregnancy [5].

Also in white blood cells (WBC) pregnancy is associated with leukocytosis, primarily related to increased circulation of neutrophils. The neutrophil count begins to increase in the second month of pregnancy and plateaus in the second or third trimester, at which time the total white blood cell counts range from 9,000 to 15,000 cells/micro liter [6]. Gestational thrombocytopenia is encountered in 7-8% of all pregnancies. Platelet counts are slightly lower during pregnancy due to accelerated destruction leading to younger, larger platelets. Most thrombocytopenia in pregnancy is due to increased destruction [7]. In view of all these modifications of the CBC test which mark the pregnancy, a follow-up of the physiological variations of the hematology parameters for pregnant woman and the determination of reference values during normal pregnancy appropriate to the target population would be of major importance. The idea is to thwart an erroneous diagnosis and a practice of useless complementary examinations, even the proposal of unfounded treatments to pregnant women. In Morocco, as in numerous countries of North Africa, the reference intervals of the CBC for pregnant women and those relative to the different trimesters of pregnancy were never established. The values used in the laboratories of medical biology taken by doctors and the above-mentioned modifications characterizing the state of pregnancy have been collected from pregnant Caucasian women or induced from studies of hematology. The preservation of their use for Moroccan pregnant woman returns their reliability questionable. With the aim of answering this deficit, we suggested the conduction of a pre-analytical and analytical study to estimate the reference values of the CBC test and the physiological modifications of the hematological parameters observed during pregnancy from a population of healthy pregnant Moroccan women from the Northwest region of Morocco. The purpose is to compare the values found by the present study to the values taken from literature and those of non-pregnant women of the same region.

## Methods

The pre-analytical phases and the analytics of our study were conducted according to the international recommendations of the IFCC-LM and the CLSI of the United States (International Federation of Clinical Chemistry - Laboratory Medicine and Clinical and Laboratory Standards Institute) relative to the establishment of the reference intervals [8, 9].

**Reference population:** In total, 3898 healthy pregnant women from 18 to 46 years old who presented themselves at the center of health Kalaa and at the service of gynecology obstetrics of the provincial hospital center of M'diq (Morocco) for prenatal were voluntarily recruited for this study from November 2014 until March 2016. The participants belonged to the middle and higher socioeconomic classes and from the different cities of the region: Tetouan, M'diq, Fnideq, and Martil. They were classified according to gestational age in three groups: (A) Women whose gestational age was less than 14 weeks of amenorrhea (1st trimester of pregnancy): 1605 pregnant women; (B) Women whose gestational age was between 14 and 28 weeks of amenorrhea (2ndtrimester of pregnancy): 1331 pregnant women; (C) Women whose gestational age was greater than 28 weeks of amenorrhea (3rd trimester of pregnancy): 962 pregnant women. The pregnant women, apparently healthy, voluntarily gave their biodata with the aid of a questionnaire and systematically underwent, before every blood drive, a medical examination with an interrogation eliminating any suspicion of diseases or visible pathologies. We excluded all the situations which could affect the CBC parameters. The criteria of exclusion in our study were of two orders: the clinical criteria: hematological, hemorrhagic or thrombotic diseases histories, viral or respiratory infections, cardiac or renal diseases, medicinal taking of nonsteroidal anti-inflammatory drugs as aspirin, high arterial blood pressure (≥ 140/90mmHg), consumption of unstable blood products in the last eight months before pregnancy, smoking or alcoholism. The biological criteria: morphological anomalies of the figurative elements of the blood observed in blood smear (hypochromia, target red blood corpuscles, plasmodium, etc). By adopting these criteria, only 3809 reference subjects were ultimately retained for the study among which 1584 women in the 1st trimester of the pregnancy, 1298 women in the 2nd trimester and 927 women in the 3rd trimester. It is necessary to note, nevertheless, that our conditions of study did not exclude patients presenting an iron deficiency and\or affected by thalassemia/hemoglobin diseases. Every subject participating in the study gave their consent freely according to the ethical standards. This study was approved by the Regional Health Committee of the Tangier-Tetouan-Al Hoceima region. With the aim of constituting a control group, the reference intervals of the CBC were established for 7035 voluntary non-pregnant women, from 18 to 50 years old, premenopausal (chosen according to the Moroccan law of blood donation); always belonging to the northwest region of Morocco. These control subjects completed a questionnaire and benefited systematically, before every donation, from a medical examination with a pre-donation interrogation eliminating any suspicion of diseases or visible pathologies. The same pre-analytical and analytical conditions used for the pregnant women were respected concerning the non-pregnant women (control group).

**Blood sampling:** In our study, we followed the standard protocol of taking and the preparation of blood samples to minimize the interpersonal variability. For every pregnant woman, the blood samples were withdrawn from the antecubital vein, in system BD Vacutainer^®^ tubes (13×75 mm) of 5ml containing an anticoagulant the K3-EDTA. The CBC test was performed the same day within 2 hours of collection.

**Hematological analysis:** A complete blood count (CBC) and differential was performed on the blood sample using Sysmex KX-21N, an automated 3-part differential hematology analyzer (Sysmex Corporation Kobe, Japan) at the laboratory of hematology of the hospital Mohamed VI of M'diq. Standardization, calibration of the instrument, and processing of the samples were done according to the manufacturer's instructions. The machine automatically dilutes whole-blood sample of 50 ml in the CBC/Differential mode, lyses and enumerates white blood cells (WBC), red blood cells (RBC), hemoglobin concentration (Hb), pack cells volume (PCV), platelets, lymphocytes, neutrophils and red blood cell indices (MCV, MCH & MCHC). It however does not count for eosinophils, monocytes and basophils counts. Therefore, a manual differential count was done on well prepared thin blood films colored by the May-Grünwald-Giemsa (MGG). The parameters studied in the optical microscope were: 1) The RBC morphology to detect possible corpuscular anomalies. 2) The leucocyte parameter was determined, double-blind, by two different operators. Each of them established the percentage of the different leucocyte populations (NEU, EOS, BAS, MON and LYM) on 200 leucocyte elements. In case of differences of more than 5 cells for a leucocyte population, formulas were double-checked by two other readings (the same operators). The final formula was established from the average of both formulas. The values absolved from the NEU, EOS, BAS, LYM and MON, expressed in 109/L, were deducted from the leucocyte numeration measured by the automate. 3) Platelets study to research morphological anomalies or platelet aggregates.

**Statistical analysis:** The data were analyzed by means of the software SPSS 20.0 (Inc, Chicago, Il). The study of the distribution of variables was made by the test of Kolmogorov-Smirnov. The quantitative variables were expressed by the median, the standard deviation and the percentiles 2.5th and 97.5th were used to limit the reference intervals. The qualitative variables were expressed in staff and percentage. The comparison of the quantitative variables was made by using the test of Mann-Whitney for two groups and the test of Kruskall-Wallis for more than two groups with complement by a correction by the test of Benferroni when the difference was significant. The comparison of the qualitative variables was made by using the test of Khi-2. A difference is considered as statistically significant if p < 0.05.

## Results

**Characteristics of the reference population:** From a total of 3898 voluntary pregnant women, 3809 were retained to establish the population of reference distributed by 1584 women in the 1st trimester of pregnancy, 1298 women in the 2nd trimester and 927 women in the 3rd trimester. Samples excluded from the study were for the following reasons: Morphological anomalies of erythrocyte on blood smears: 1st trimester of pregnancy: Hypochromia (02 subjects), anisocytosis (18 subjects) and target RBC (01 subject); 2nd trimester of pregnancy: Anisocytosis (28 subjects) and target RBC (05 subjects); 3rd trimester of pregnancy: Anisocytosis (30 subjects) and target RBC (05 subjects). The mean age of the pregnant women was 28.54 ± 6.13 years while that of the non-pregnant women (control group) was of 28.86 ± 8.26 years. Table 1 shows the means as well as the standard deviations of the age and body mass index according to the different trimesters of the pregnancy. Table 2 represents the distribution of the non-pregnant women according to the trimesters of the pregnancy and the provinces; whereas those of the pregnant women according to iron supplementation are represented in the Table 3. According to the Table 2, the rate of the studied pregnant women from the prefecture of M'diq-Fnideq was 65.7% while those of the province of Tetouan were 34.3%. Table 3 shows that the rate of iron supplemented pregnant women is very reduced (13.9%) compared with the non-iron supplemented pregnant women (86.1%).

**Table 1:** Means and standard deviations of age and body mass index for pregnant women analyzed according to the trimesters of pregnancy

	Trimester: mean ± standard deviation					
	Overall (n=3908)	First trimester (n=1584)	Second trimester (n=1298)	Third trimester (n=927)
Age (years)	28.54 ± 6.13	28.29 ± 6.22	28.52 ± 6.63	28.98 ± 5.16
Body Mass Index (kg/m^2^)	26.76 ± 3.73	26.65 ± 3.21	25.80 ± 3.82	28.30 ± 3.98

**Table 2:** Distribution of the pregnant women population according to the trimesters of the pregnancy and the provinces

District	Overall		First trimester		Second trimester		Third trimester	
	n	%	N	%	n	%	n	%
Tetouan	1306	34.3	566	35.7	463	35.7	277	29.9
M'diq-Fnideq	2503	65.7	1018	64.3	835	64.3	650	70.1
Overall	3809	100	1584	100	1298	100	927	100

**Table 3:** Distribution of the pregnant women population according to iron supplementation

Iron supplements(60-80 mg ferrous iron/day)	Overall		First trimester		Second trimester		Third trimester	
	n	%	N	%	n	%	n	%
Non-iron supplements	3279	86.1	1486	93.8	1110	85.5	683	73.7
Iron supplements	530	13.9	98	6.2	188	14.5	244	26.3
Overall	3809	100	1584	100	1298	100	927	100

**The complete blood count test:** A) blood count parameters for pregnant women and non-pregnant women (control): the means, standard deviations, medians and reference intervals of the CBC parameters for the pregnant women and non-pregnant women are presented in Table 4. A significant difference between the pregnant women and the control group was noted (p < 0.05) with regard to all the studied hematological parameters: RBC, HGB, HCT, MCV, MCH, MCHC, WBC, LYM, MON, NEU, EOS, BAS, PLT and MPV. We noted that mean erythrocytes parameters RBC, HGB, HCT and MCV for non-pregnant women were higher than those of the pregnant women: RBC 4.51 × 1012/L (3.86 - 5.20 × 1012/L) for non-pregnant women versus 4.07 × 1012/L (3.29 - 4.85 × 1012/L) for pregnant women (p < 0.001), HGB 13.01 g/dL (11 - 14.8 g/dL) for non-pregnant women versus 11,80 g/dL (9.4 - 13.7 g/dL) for pregnant women (p < 0.001), HCT 38.61% (33.5 - 43.9 %) for non-pregnant women versus 34.73% (28.6 - 40.5%) for pregnant women (p < 0.001) and MCV 85.82 fL (75.1 - 94.7 fL) for non-pregnant women versus 85.28 fL (74 - 96 fL) for pregnant women (p < 0.001). The mean of the MCH and the MCHC was superior for pregnant woman when compared with non-pregnant women: MCH 28.93 pg (24 - 32.3 pg) for non-pregnant women versus 29.05 pg (23.7 - 33.2 pg) for pregnant women (p = 0.01) and MCHC 33,69 g/dL (31.2 - 36 g/dL) for non-pregnant women versus 34.02 g/dL (31.2 - 36.5 g/dL) for pregnant women (p < 0.001). The mean of the total leukocytes and the NEU was higher for pregnant woman than for non-pregnant women: WBC 8.18 × 109/L (4.6 - 13.0 × 109/L) for pregnant women versus 7.12 × 109/L (4.1 - 10.7 × 109/L) for non-pregnant women (p < 0.001) and NEU 5.31 × 109/L (2.2 - 9.7 × 109/L) for pregnant women versus 4.08 × 109/L (1.8 - 7.0 × 109/L) for non-pregnant women (p < 0.001). For platelet numeration, the mean value for pregnant women was lower than that observed for non-pregnant women. It was 234.89 × 109/L (141 - 377 × 109/L) for pregnant women and 243 × 109/L (150 - 378 × 109/L) for non-pregnant women, respectively (p < 0.001). In the same way, the mean of the MPV was lower for pregnant women compared to non-pregnant women: 10.89 fL (8.9 - 13.5 fL versus 11.2 fL (9 - 13.7fL, respectively (p < 0.001).

**Table 4:** Comparison of the means, standard deviations, medians and reference intervals of the blood count parameters for pregnant women and non-pregnant women

Pregnant women (n=3908)						Non Pregnant women (control) (n=7035)					P value
				Reference interval					Reference interval		
Hematological parameters	Mean	SD	Median	Percentile 2.5	Percentile 97.5	Mean	SD	Median	Percentile 2.5	Percentile 97.5
RBCx1012/L	4.07	0.40	4.1	3.29	4.85	4.51	0.35	4.5	3.86	5.20	<0.001
HGB (g/dL)	11.80	1.06	11.9	9.4	13.7	13.01	0.94	13	11	14.8	<0.001
HCT (%)	34.73	3.10	34.9	28.6	40.5	38.61	2.72	38.6	33.5	43.9	<0.001
MCV (fL)	85.28	5.42	85.5	74	96	85.82	4.92	86.2	75.1	94.7	<0.001
MCH (pg)	29.05	2.35	29.3	23.7	33.2	28.93	2.07	29.2	24	32.3	0.01
MCHC (g/dL)	34.02	1.45	34.1	31.2	36.5	33.69	1.22	33.7	31.2	36	<0.001
WBCx109 /L	8.18	2.18	7.9	4.6	13.0	7.12	1.68	7	4.1	10.7	<0.001
LYMx109/L	2.17	0.60	2.1	1.2	3.6	2.33	0.65	2.2	1.2	3.8	<0.001
MONx109 /L	0.53	0.29	0.5	0.1	1	0.59	0.30	0.5	0.2	1.2	<0.001
NEUx109/L	5.31	1.96	5.1	2.2	9.7	4.08	1.35	3,9	1.8	7	<0.001
EOSx109/L	0.14	0.15	0.1	0	0.4	0.13	0.17	0.1	0	0.5	<0.001
BASx109/L	0.02	0.03	0	0	0.1	0.01	0.03	0	0	0.08	<0.001
PLTx109/L	234.89	60.42	227	141	377	243.50	58.41	237	150	378	<0.001
MPV (fL)	10.89	1.22	10.8	8.9	13.5	11.20	1.19	11.2	9	13.7	<0.001

SD, standard deviation; RBC, red blood cell; HGB, hemoglobin; HCT, hematocrit; MCV, mean corpuscular volume; MCH, mean corpuscular hemoglobin; MCHC, mean corpuscular hemoglobin concentration; WBC, white blood cell; LYM, lymphocytes; MON, monocytes; NEU, neutrophils; EOS, eosinophils; BAS, basophils; PLT, platelets; MPV, mean platelet volume.

Mann-Whitney U-test for nonnormally distributed parameters was done between pregnant women and non-pregnant women: All hematological parameters showed significant differences between pregnant and non-pregnant women.

P < 0.05 was considered as statistically significant.

B) Blood count parameters for pregnant women according to the trimesters of the pregnancy: the mean, standard deviations, medians and reference intervals of the CBC parameters for pregnant women according to the trimesters of pregnancy are presented in Table 5. The study showed variations of the erythrocytes, leukocytes and platelet parameters according to the trimesters of pregnancy. A significant difference in the values of RBC, HGB, HCT, MCV, MCH, MCHC according to the trimesters was noted (p < 0.001). Additionally, the mean values of the RBC, HGB, HCT and MCHC showed a progressive decrease with gestational age, particularly in the 3rd trimester of pregnancy. The mean value the RBC was of 4.21 ± 0.36 × 1012/L (3.49 - 4.91 × 1012/L), 4.02 ± 0.39 × 1012/L (3.36 - 4.82 × 1012/L) and 3.92 ± 0.41 × 1012/L (3.19 - 4.78 × 1012/L) in 1st, 2nd and 3rdtrimester of pregnancy, respectively. The mean value of the HGB was 12.23 ± 0.93 g/dL (10 - 13.9 g/dL), 11.68 ± 0.96 g/dL (9.6 - 13.6 g/dL) and 11.22 ± 1.06 g/dL (9.1 - 13.4 g/dL) in the 1st, 2ndnd and 3rd trimester of pregnancy, respectively. The mean value of the HCT was 35.87 ± 2.83% (29 - 40.9%), 34.44 ± 2.94% (28.6 - 39.9%) and 33.20 ± 2.98% (27.34 - 39.3%) in the 1st, the 2nd and the 3rd trimester of pregnancy, respectively. Finally, the mean value of the MCHC was 34.13 ± 1.43 g/dL (31.3 - 36.6 g/dL), 34.01 ± 1.47 g/dL (31.2 - 36.6 g/dL) and 33.82 ± 1.45 g/dL (30.8 - 36.2 g/dL) in the 1st, 2nd and 3rd trimester of pregnancy, respectively.

**Table 5:** Comparison of the means, standard deviations, medians and reference intervals of the blood count parameters for pregnant women studied according to the trimesters of pregnancy

Trimester	First trimester (n=1584)					Second trimester (n=1298)					Third trimester (n=927)					P value
Hematological parameters				Reference interval					Reference interval					Reference interval		
	Mean	SD	Median	Percentile 2.5	Percentile 97.5	Mean	SD	Median	Percentile 2.5	Percentile 97.5	Mean	SD	Median	Percentile 2.5	Percentile 97.5	
RBCx1012/L	4.21	0.36	4.23	3.49	4.91	4.02	0.39	4.02	3.26	4.82	3.92	0.41	3.93	3.19	4.78	<0.001	
HGB (g/dL)	12.23	0.93	12.3	10	13.9	11.68	0.96	11.7	9.6	13.6	11.22	1.06	11.3	9.1	13.4	<0.01
HCT (%)	35.87	2.83	36.2	29.8	40.9	34.44	2.94	34.5	28.6	39.9	33.20	2.98	33.1	27.34	39.3	<0.001
MCV (fL)	85.21	5.02	85.6	74.4	94.9	85.73	5.58	85.7	74.7	97.7	84.77	5.79	85	72.8	96.1	<0.001
MCH (pg)	29.14	2.19	29.3	24.2	32.9	29.17	2.36	29.4	24.0	33.3	28.71	2.56	28.9	23	33.4	<0.001
MCHC (g/dL)	34.13	1.43	34.2	31.3	36.6	34.01	1.47	34.1	31.2	36.6	33.82	1.45	34	30.8	36.2	<0.001
WBCx109 /L	7.52	1.78	7.5	4.5	11.6	8.03	2.02	7.9	4,6	12.6	9.53	2.39	9.5	5.3	14.3	<0.001
LYMx109/L	2.16	0.56	2.1	1.2	3.4	2.15	0.60	2.1	1.2	3.6	2.20	0.66	2.1	1.1	3.8	<0.001
MONx109 /L	0.49	0.28	0.4	0.1	1	0.53	0.28	0.5	0.2	1	0.62	0.29	0.6	0.1	1	<0.001
NEUx109/L	4.68	1.59	4.5	2.1	8.2	5.18	1.80	5.1	2.2	9.2	6.56	2.15	6.5	3	11	<0.001
EOSx109/L	0.16	0.15	0.1	0	0.4	0.14	0.14	0.1	0	0.4	0.09	0.13	0	0	0.4	<0.001
BASx109/L	0.01	0.03	0	0	0.09	0.02	0.04	0	0	0.1	0.02	0.04	0	0	0.1	<0.001
PLTx109/L	235.85	57.61	229	145	374	229.90	58.44	224	140	364	240.25	67.02	231	139	398	<0.001
MPV (fL)	10.97	1.20	10.8	8.9	13.7	10.98	1.23	10.9	8.9	13.5	10.64	1.20	10.6	8.9	13.2	<0.001

SD, standard deviation; RBC, red blood cell; HGB, hemoglobin; HCT, hematocrit; MCV, mean corpuscular volume; MCH, mean corpuscular hemoglobin; MCHC, mean corpuscular hemoglobin concentration; WBC, white blood cell; LYM, lymphocytes; MON, monocytes; NEU, neutrophils; EOS, eosinophils; BAS, basophils; PLT, platelets; MPV, mean platelet volume.

Mann-Whitney U-test for non-normally distributed parameters was done between trimesters: All hematological parameters showed significant differences between first, second, and third trimester.

P < 0.05 was considered as statistically significant.

Comparison between trimesters of pregnancy: Comparing the three groups two by two using a post-hoc test shows that there is a statistically significant difference between the three groups taken two by two

For leukocyte lineage, the study revealed a progressive increase of the total number of WBC and the NEU with gestational age, especially in the 3rd trimester of pregnancy. Indeed, the mean value of the WBC was 7.52 ± 1.78 × 109/L (4.5 - 11.6 × 109/L), 8.03 ± 2.02 × 109/L (4.6 - 12.6 × 109/L) and 9.53 ± 2.39 × 109/L (5.3 - 14.3 × 109/L), in the 1st, 2nd and 3rd trimester of pregnancy, respectively. The mean value of the NEU was 4.68 ± 1.59 × 109/L ( 2.1 - 8.2 × 109/L), 5.18 ± 1.80 × 109/L ( 2.2 - 9.2×109/L) and 6.56 ± 2.15 × 109/L (3.0 - 11.0 × 109/L) in the 1st, 2nd and 3rd trimester of pregnancy, respectively. This increase was statistically significant between the 1st, 2nd and 3rd trimester of the pregnancy (p < 0.001). The mean value of platelet numeration according to the trimesters of pregnancy was superior in the 3rd trimester, 240.25 ± 67.02 × 109/L (139 - 398 × 109/L), compared with the 1st trimester, 235.85 ± 57.61 × 109/L (145 - 374 × 109/L), and the 2ndtrimester of the pregnancy, 229.90 ± 58.44 × 109/L (140-364 × 109/L). According to trimesters, this difference was statistically significant (p < 0.001). On the other hand, the mean value of the MPV according to the trimesters of the pregnancy was superior in the 2nd trimester, 10.98 ± 1.23 × 109/L (8.9 - 13.5 × 109/L), compared with the 1st trimester, 10.97 ± 1.20 × 109/L (8.9 - 13.7 × 109/L) and the 3rd trimester of pregnancy, 10.64 ± 1.20 × 109/L (8.9 - 13.2 × 109/L). This difference according to trimesters of the pregnancy was statistically significant (p < 0.001).

## Discussion

Pregnancy is characterized by a deep modification of physiological functions of the pregnant woman's body. Indeed, during the pregnancy, there is a considerable increase of the metabolic needs, as well as modifications of the hormonal balance. These phenomena provide enough justification for the hematological disorders. This study was done to estimate the reference values of CBC parameters as well as estimate the hematological modifications that arise during pregnancy in healthy women from the Northwest region of Morocco. A follow-up of the different parameters of the CBC test during the trimesters of pregnancy was also conducted. According to the results of this study, we noticed that the rate of HGB was significantly lower for pregnant women, 11.80 ± 1.06 g/dL (9.4 - 13.7g/dL), in comparison to the control group (non-pregnant women), 13.01 ± 0.94 g/dL (11 - 14.8 g/dL), and that this rate decreased gradually with gestational age: HGB was 12.23 ± 0.93 g/dL (10 - 13.9 g/dL), 11.68 ± 0.96 g/dL (9.6 - 13.6 g/dL) and 11.22 ± 1.06 g/dL (9.1 - 13.4 g/dL) in the 1st, 2nd and 3rdtrimester of pregnancy, respectively. Our result are in agreement with that of the study of Geetanjali et al which revealed a HGB of 10.03 ± 1.12 g/dL for pregnant women versus 11.2 ± 1.16 g/dL for non-pregnant women. This study also showed a HGB in the 1st trimester of pregnancy of 10.48 ± 0.89 g/dL, 10.66 ± 1.04 g/dL in the 2nd trimester and 10.02 ± 1.26 g/dL in the 3rd trimester [10]. This reduction phenomenon of the HGB for pregnant women can be correlated, on the one hand, to the physiological anemia induced by progressive hemodilution (the plasmatic volume increases more quickly than the erythrocyte mass) [11] and on the other hand, to increased need of vitamins and minerals for fetal hematopoiesis (iron, vitamin B12, folic acid) [2]. This is physiological modifications which occur in all pregnant women to compensate for the needs associated with the fetus and its environment [11].

The mean values of the HGB obtained in our population respect the threshold established by the CDC in the United States and that of the WHO. The lower values of HGB corresponding to the lower limits of the reference intervals in our population with regard to the literature and to the Caucasian population (longitudinal study performed for 434 Danish pregnant women) [12, 13] (Table 6), could be explained by a higher frequency of iron deficiency. On a national scale, iron-deficiency anemia is a health problem in Morocco and affects more than a third of the Moroccan population with an ascendancy in pregnant women (37.2%) and women old enough to procreate (33%) [14]. As for the international scale, according to the WHO, anemia in the world affects 30.2% of women old enough to procreate and 41.8% of pregnant women [15]. Besides, these lower values of HGB corresponding to our study could be also owed to the reduced number of iron supplemented pregnant women (13.9%). The necessity to or not to bring systematic iron supplementation, in the beginning of pregnancy, as a preventative measure for women who are neither anemic nor iron deficient represents a controversial central question. The problem is also discussed in terms of dose to be administered when beginning treatment. The studies of the primary and secondary objectives to achieve such supplementation are also controversial. The recommendations of the WHO (1989) advise administering all pregnant women 60 mg of iron a day in addition to 250 mg of folic acid in regions where prevalence of ferric deficiency is lower than 20%, and the doubling of these doses in regions where prevalence is higher [16]. Besides iron deficiency, hemoglobin diseases could be responsible in the low levels of the lower limits of the HGB of our study because Morocco is a part of main Mediterranean countries affected by thalassemia, it is ranked 28th in the world and prevalence of the carriers of the beta-thalassemia is in the order of 3% [17]. Deficiencies in vitamins and minerals, in particular iron, the unavailability of iron supplementation for exposed women, socioeconomic level, lack of good sanitary and nutritional education, as well as a higher index of gestation with, consequently, more gestational losses, would represent factors to explain these decreased lower limits. To re-emphasize our study was unable to eliminate from our population of study the subjects presenting an iron deficiency or those affected by hemoglobin diseases. In developed countries, clinical trials used discriminatory values of the HGB going from 10.0 to 11.4 g/dL [18, 19].

**Table 6:** Comparison of the reference intervals of the erythrocytes index of our study with those found in the literature (longitudinal study performed for 434 Danish pregnant women)

	Our study (with and without iron supplements)		Literature (with iron supplements) [12,13]	
Red cell indices	Second trimester	Third trimester	Second trimester 18weeks	Third trimester32 weeks
RBCx1012/L	4.02(3.26 –4.82)	3.92(3.19 – 4.78)	3.93 (3.43–4.49)	3.86 (3.38–4.43)
HGB (g/dL)	11.68(9.6-13.6)	11.22(9.1 – 13.4)	11.9 (10.6–13.3)	11.9 (10.4–13.5)
HCT (%)	34.44(28.6 – 39.9)	33.20(27.34 – 39.3)	35 (31–39)	35 (31–40)
MCV (fL)	85.73(74.7 – 97.7)	84.77(72.8 – 96.1)	89 (83–96)	91 (85–97)
MCH (pg)	29.17(24.0 – 33.3)	28.74(23.0 – 33.4)	30 (27–33)	30 (28–33)
MCHC (g/dL)	34.01(31.2 – 36.6)	33.82(30.8 – 36.2)	34 (33–36)	34 (33–36)

Mean and reference ranges (2.5th – 97.5th centiles). RBC, red blood cell; HGB, hemoglobin; HCT, hematocrit; MCV, mean corpuscular volume; MCH, mean corpuscular hemoglobin; MCHC, mean corpuscular hemoglobin concentration

With regards to the mean values of the RBC and those of the HCT or PCV (packed cell volume), they decreased significantly for pregnant women compared to non-pregnant women (p < 0.05). These results are similar to those of Geetanjali et al. which produced means of RBC of 4.09 ± 0.37 × 1012/L for pregnant women versus 4.26 ± 0.26 × 1012/L for non-pregnant women and means of HCT of 34.89 ± 9.28 % versus 39.88 ± 3.12% for pregnant and non-pregnant women, respectively [10]. The study of Somendra Kumar Dhariwal et al. also brings concurring results because mean RBC was found to be of 4.23 ± 0.62 × 1012/L for pregnant women versus 4.29 ± 0.55 × 1012/L for non-pregnant women and means of the HCT were 32.52 ± 5.08% for pregnant women versus 34.6 ± 15.3% for non-pregnant women [20]. We noticed a progressive decrease in the rate of RBC and the HCT with gestational age. This report is consolidated by the study of Mohamed et al. which produced means of the RBC of 4.31 ± 0.49 × 1012/L in the 1st trimester, 4.06 ± 0.48 × 1012/L in the 2ndtrimester and 4.01 ± 0.48 × 1012/L in the 3rd trimester of pregnancy [2], and by the study of Geetanjali et al. which produced means of the HCT of 37.51 ± 2.6% in the 1st trimester, 32.88 ± 2.96% in the 2nd trimester and 33.7 ± 3.27% in the 3rd trimester [10]. The reduction in the rate of the HCT, accompanied by a decrease in the number of the RBC during pregnancy could be associated either with the increase of the plasmatic volume while the pregnancy progresses causing a hemodilution, or from the hormonal changes which increase fluid retention and iron deficiency [20, 21]. The lower limit of the reference intervals of the HCT of studied pregnant women was 27,34% at the 3rd trimester of the pregnancy. This value is lower than that of the Milman et al study where the lower limit of the HCT was 31% in the 2nd and 3rd trimester of pregnancy [12]. The WHO defines the threshold of anemia for pregnant woman when the rate of hemoglobin is lower in 11 g/dL and that of the HCT lower than 33% at any time of the pregnancy [5]. For practical clinical use, other authors, in previous studies led in industrialized countries, suggested a rate of HCT lower than 30 % as the discriminatory value of anemia for pregnant woman [19].

The mean values of the erythrocytes index MCV, MCH and MCHC showed a significant difference (p < 0.05) between pregnant women and control, and between the different trimesters of pregnancy (p < 0.05). These results do not coincide with those of the Geetanjali et al study where the mean values of MCH and MCHC did not show any significant difference between the pregnant women and the control (p > 0.05) while the difference was statistically significant for the MCV (p < 0.05) [10]. In our study, the reference intervals of MCV, MCH and MCHC for pregnant women were very close to those of non-pregnant women, but the lower limits of the reference intervals obtained for pregnant women were lower than that of the literature and that of a study of the Caucasian population which include only the iron supplemented pregnant women (20-80 mg of ferrous iron/day) [12, 13] (Table 6). These lower limits could be explained by the high frequency of iron-deficiency anemia in our population. Indeed, in Morocco 48% of the pregnant women present anemia iron deficiency [14]. During normal pregnancy, there is an increase of leukocytosis bound mainly to an increase of the number of NEU [1]. This leukocytosis is due to the physiological stress led by the state of pregnancy and an increase in the number of NEU is probably a homeostatic answer to an apoptosis of the altered NEU expressed during pregnancy [10]. Our results show a significant increase (p < 0.001) of the mean number of the total WBC for pregnant women compared with non-pregnant women. This rate evolved as gestational age progresses following the results obtained by the Pughikumo et al study: 6.38 ± 1.76 × 109/L in the 1st trimester, 6.81 ± 1.52 × 109/L in the 2nd trimester and 7.36 ± 1.49 × 109/L in the 3rd trimester [22]. In the same way, Akinbami et al relate 7.31 ± 2.38 × 109/L in the 1st trimester, 7.88 ± 2.33 × 109/L in the 2nd trimester and 8.37 ± 2.15 × 109/L in the 3rd trimester [23]. In a Crocker IP et al study, the same tendency was confirmed as the mean number of the total WBC in the 1st trimester was of 7.32 ± 0.68 × 109/L, 7.81 ± 0.71 × 109/L in the 2ndtrimester and 10.24 ± 1.30 × 109/L in the 3rd trimester [24]. At the same time as the increase of total WBC, the mean value of the number of NEU was significantly higher for pregnant women compared with non-pregnant women (p < 0.001). We noticed that the progressive increase of NEU was statistically significant according to the gestational age (p < 0,001). Our results are in agreement with those of Okpokam et al study who show a value of 4.7 ± 2.80 × 109/L in the 1st trimester, 5.1 ± 2.41 × 109/L in the 2nd trimester and 6.0 ± 5.94 × 109/L in the 3rd trimester [25].

The mean value of the lymphocytes number was significantly decreased for pregnant women compared with non-pregnant women (p < 0.001). This result is in agreement with that of a recent study performed in Turkey by Gökçen Örgül et al The mean number of LYM before pregnancy was 2049.07 ± 758.69 cells/μL and during pregnancy 1850.93 ± 501.86 cells/μL. This decrease is probably due to the pregnancy and related hormonal changes which would have a negative impact on the blood count of the total lymphocytes [26]. The mean values of BAS and EOS were significantly superior for pregnant women compared with non-pregnant women (p < 0.001) while those of MON were significantly lower for pregnant women compared with non-pregnant women (p < 0.001). In our study, the comparison of the mean values of BAS, EOS and MON between the 1st, 2nd and 3rd trimester of the pregnancy, showed a statistically significant difference (p < 0.001). These results disagree with those of Okpokam et al who show that there were no statistical difference of the EOS and MON between the 1st, 2nd and 3rd trimester of pregnancy (p > 0.05) [25]. The reference intervals of the EOS, BAS, MON and LYM which we looked for in this work for pregnant women were close to those of non-pregnant women, except for the total WBC and the NEU where the superior limits of the reference intervals obtained for pregnant women were higher with regard to those found for non-pregnant women. Our reference intervals of leukocytes parameters diverge in their majority compared to the intervals published in the study of Balloch and Cauchi (Table 6 (suite)), performed for an Australian population composed of 11,210 pregnant women by a Coulter Counter S PLUS device. This study confirmed the increase of leukocytosis and polynucleosis during pregnancy until the 34th week of pregnancy and their small decrease [27]. Concerning platelet numeration, the mean value of the PLT number for pregnant women was significantly lower with regard to that observed in non-pregnant women (p < 0.001). This result is similar to that of the study of Obeagu Emmanuel Ifeanyi et al which produced a mean value of PLT of 122 ± 3.4 × 109/L for pregnant women and 198.5 ± 5.6 × 109/L for non-pregnant women (p < 0.05) [28]. Besides, the mean value of platelet numeration was superior for women in the 3rd trimester of pregnancy compared to those in the 1st and 2nd trimester; this difference was statistically significant (p < 0.001). This result did not suit the study of James et al where the mean value of the PLT was lower at the 3rd trimester of the pregnancy (234.15 ± 67.67 × 109/L) compared with to the 1st trimester (280.55 ± 64.40 × 109/L) and 2nd trimester (250.32 ± 67.95 × 109/L) [29].

**Table 6 (suite):** Comparison of the reference intervals (2.5th – 97.5th percentiles) of leukocytes and platelet parameters of our study with those found in the Australian study of Balloch and Cauchi (1993)

	Our study			Balloch and Cauchi [27]		
Leucocyte and platelet parameters	First trimester	Second trimester	Third trimester	First trimester	Second trimester	Third Trimester
WBCx109 /L	4.5-11.6	4.6-12.6	5.3-14.3	5.7-13.6	6.2- 14.8	5.9-16.9
LYMx109/L	1.2-3.4	1.2-3.6	1.1-3.8	1.1-3.5	0.9-3.9	1.0-3.6
MONx109 /L	0.1-1.0	0.2-1	0.1-1	0.1-1.1	0.1-1.1	0.1-1.4
NEUx109/L	2.1-8.2	2.2-9.2	3.0-11.0	3.6-10.1	3.8-12.3	3.9-13.13
EOSx109/L	0-0.4	0-0.4	0-0.4	0-0.6	0-0.6	0- 0.6
BASx109/L	0-0.09	0-0.1	0-0.1	0-0.1	0- 0.1	0-0.1
PLTx109/L	145-374	140-364	139 - 398	174-391	17 1-409	155-429

WBC, white blood cell; LYM, lymphocytes; MON, monocytes; NEU, neutrophils; EOS, eosinophils; BAS, basophils; PLT, platelets

In our study, the lower limits of the reference values of the platelet numeration were lower for pregnant women (141 - 377 × 109/L) than for non-pregnant women (150 - 378 × 109/L). This lower limit was less than 150 × 109/L for women in the 1st, 2nd and 3rd trimester of pregnancy. On the other hand, the lower limits of the platelet numeration of the study of Balloch and Cauchi were greater than 150 × 109/L with, respectively, 174 - 391 × 109/L, 171 - 409 × 109/L and 155 - 429 × 109/L, for the 1st, 2nd and 3rd trimester of pregnancy (Table 6 (suite)) [27]. The discovery of a thrombopenia during pregnancy called gestational thrombopenia (PLT number lower than 150 × 109/L) is, indeed, a relatively frequent situation and usually asymptomatic. A rate of PLT between 70 000 and 150 000/mm3 was described by Burrows and Kelton in approximately 8% of the pregnancies, this rate normalizes mainly in four weeks after the childbirth [30, 31]. This report could be attributed, on the one hand, to dilution by an increase of the plasmatic volume and on the other hand, to a compensatory phenomenon due to maximal platelet destruction during the third trimester as shown by the increase of the mean platelet volume. This gestational thrombopenia comes along with a platelet hyperreactivity to diverse agents aggregating, bound to a greater synthesis of thromboxane A2 [32, 33]. The mean value of the MPV was significantly higher for non-pregnant women with regard to the pregnant women (p < 0.001). We can deduct from this result that no increase of MPV of pregnant women took place in our study. The reference intervals of the MPV were more similar between pregnant and non-pregnant women, in the three trimesters of pregnancy. By way of synthesis, we noticed that the physiological variation of the hematological parameters during pregnancy makes difficult the definition of the "normal" hematological reference intervals for pregnant women. We suppose that the iron-deficiency anemia during pregnancy is not a physiological situation. In future studies, the determination of reference intervals for "normality" for pregnant Moroccan women should be conducted in pregnant women supplemented with iron.

## Conclusion

The present work constitutes a first attempt for the establishment of reference values of the CBC for a population of healthy pregnant women living in the Northwest region of Morocco. The study acknowledges the need to procure other data from different regions of the country. The results of this work consolidate the idea that pregnancy is a phenomenon which induces a physiological change of certain parameters of the blood count and it is therefore of great interest to take into account these modifications for optimal maternal and fetal medical care.

### What is known about this topic

- The parameters of CBC are influenced by many factors like pregnancy;
- During normal pregnancy, changes occur and can be observed in hematological indices such as red blood cell (RBC) count, hemoglobin (HB) concentration "physiologic anemia", platelet (PLT) count, and white blood cell (WBC) count.

### What this study adds

- Provide data of hematological reference values in a healthy population of pregnant women in the Northwest of Morocco. These values should prove useful for diagnostic and research purposes;
- Conclude that healthy pregnancy has the tendency to alter some hematological indices. Therefore, Clinicians' familiarity with these pregnancy related physiological changes in the hematologic system will encourage an optimal management of pregnancies;
- The findings of this study reinforce the need for supplementation and to procure other data from different regions of the country.
